# Supplementary material for: Vitamin D Modified DSS-Induced Colitis in Mice via STING Signaling Pathway
Source: Biology (Basel). 2025 Jun 18;14(6):715. doi: 10.3390/biology14060715 (PMC12190092; doi:10.3390/biology14060715)
Supplement: Supplementary file 1 [file biology-14-00715-s001.zip › Supplementary Materials S1: 16s-report of gut microbiota/Groups/diff_analysis/TukeyHSD/Tukey_KEGG.pdf]

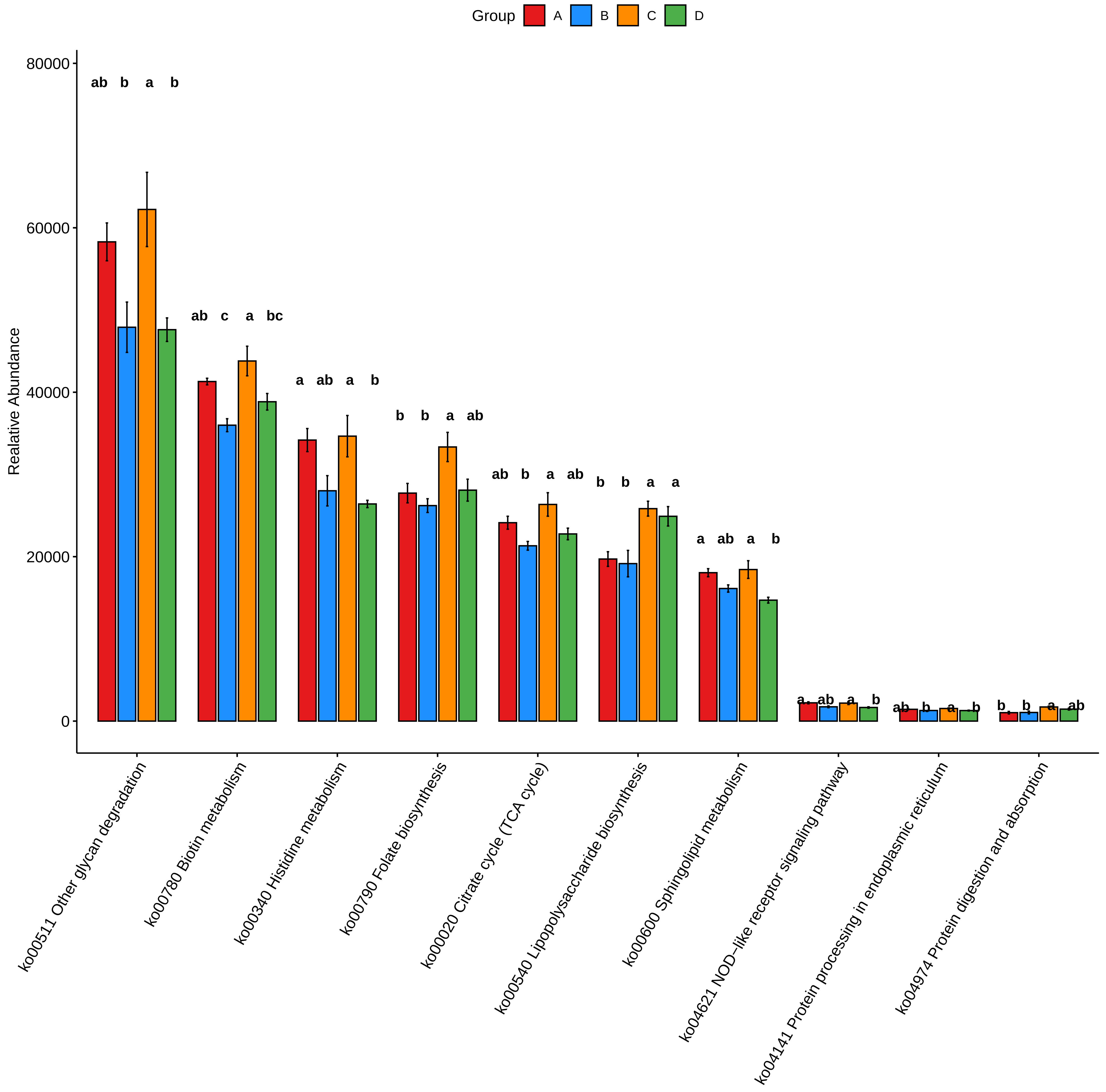

Group
A
B
C
D
Realative Abundance
80000
60000
40000
20000
0
ab b a b
ab c a bc
a ab a b
b b a ab
ab b a ab
b b a a
a ab a b
a ab a b
ab b a b
b b a ab
ko00511 Other glycan degradation
ko00780 Biotin metabolism
ko00340 Histidine metabolism
ko00790 Folate biosynthesis
ko00020 Citrate cycle (TCA cycle)
ko00540 Lipopolysaccharide biosynthesis
ko00600 Sphingolipid metabolism
ko04621 NOD−like receptor signaling pathway
ko04141 Protein processing in endoplasmic reticulum
ko04974 Protein digestion and absorption
